# Pullback Pressure Gradient—An Emerging Concept in Patients with Coronary Artery Disease

**DOI:** 10.31083/j.rcm2508278

**Published:** 2024-08-07

**Authors:** Muntaser Omari, Abdalazeem Ibrahem, Bilal Bawamia, Timothy Cartlidge, Alan Bagnall, Ian Purcell, Mohaned Egred, Azfar Zaman, Mohamed Farag, Mohammad Alkhalil

**Affiliations:** ^1^Cardiothoracic Centre, Freeman Hospital, NE7 7DN Newcastle-upon-Tyne, UK; ^2^Translational and Clinical Research Institute, Newcastle University, NE1 7RU Newcastle-upon-Tyne, UK

**Keywords:** coronary artery disease, physiology, pressure wire, pullback pressure gradient, diffuse, focal, ischemia, symptoms, clinical outcomes

## Abstract

Fractional flow reserve fractional flow reserve (FFR)-guided percutaneous coronary intervention (PCI) is currently recommended in the management of patients with stable coronary artery disease (CAD). Pullback pressure gradient (PPG) index is an emerging concept that provides a fully quantitative measure of the longitudinal distribution of CAD. It can be derived from FFR, as well as other non-hyperemic indices, and is a novel metric of assessing the focality or diffuseness of CAD. PPG adds a second domain to the assessment of CAD, beyond ischemia as measured by FFR, and may enable clinicians to better inform their patients about the status of their CAD but may also predict potential outcomes before revascularization. In this article, we will provide an in-depth review on the concept of PPG index and its correlation to pre and post revascularization ischemia. We will assess the relationship between PPG index and plaque characteristics and how this is translated into any difference in procedural and long-term clinical outcomes.

## 1. Introduction

Myocardial revascularisation with either percutaneous coronary intervention (PCI) or coronary artery bypass graft (CABG) surgery is indicated when there is documentation of significant obstruction to coronary blood flow associated with myocardial ischemia in patients for whom medical therapy has failed or is expected to lead to suboptimal outcomes [1]. Current guidelines recommend the use of invasive physiological assessment of coronary lesions to guide revascularization [1, 2]. This is particularly important for patients with intermediate coronary stenoses, whereby the accuracy of invasive coronary angiography alone in predicting the hemodynamic significance of coronary stenosis is less than 50% [1, 2].

Fractional flow reserve (FFR) is a well-established method to invasively assess coronary ischemia and appear to improve clinical outcomes when guiding percutaneous coronary revascularization [3, 4]. Non-invasive assessment of coronary ischemia using quantitative flow ratio (QFR) as well as invasive resting indices are also recognised methods to detect ischemia and to guide percutaneous coronary revascularization [5, 6, 7]. FFR has shown to be a useful tool in guiding treatment or for risk stratification in complex clinical as well as anatomical subsets of patients undergoing coronary revascularization. This includes patients with acute coronary syndrome (ACS), impaired left ventricle function, women, renal disease, and diabetes [8, 9, 10, 11, 12, 13]. On the other hand, patients with serial lesions may render physiological assessment of coronary lesions more challenging [14]. The interaction among serial coronary lesions is well-established in the literature and often resulted in underestimating the hemodynamic significance of coronary lesions [14, 15, 16, 17, 18, 19, 20]. A study by Kim *et al*. [14] highlighted that true FFR was lower than apparent FFR in the proximal and distal lesions; with a significant pressure step-up of a non-primary target lesion after stenting the primary target lesion [14, 19, 20]. Therefore, better characterization of coronary anatomy would add further insights into the role of ischemia assessment in patients with coronary artery disease (CAD). An emerging concept has recently been proposed to allow better evaluation of coronary anatomy. Pullback pressure gradient (PPG) index was introduced to provide a quantitative metric of patterns of CAD [17, 21]. This review article provides an overview of this concept, its relation to ischemia, and potential future role in the management of patients with CAD.

## 2. Patterns of Coronary Artery Disease

The anatomical distribution of atherosclerotic CAD was early recognized using both invasive and non-invasive imaging modalities [22, 23]. Terms such as focal or diffuse CAD are well-established in clinical practice and have been linked to patients clinical outcomes. When compared head-to-head, the prevalence and the extent of CAD patters were not similar using various imaging modalities [22, 23]. In fact, invasive coronary angiography systematically underestimated the presence and distribution of CAD when compared to computed tomography (CT) and intra-vascular ultrasound (IVUS) [22, 23]. Diffuse CAD was detected on IVUS in patients with angiography normal coronary arteries [20, 24]. Overall, the accuracy of using coronary angiography in classifying patterns of CAD into focal, diffuse, or combined was very modest with significant inter-observer variability when assessing advanced coronary stenoes [21].

There is a well-established relationship between patterns of CAD and clinical outcomes following coronary revascularization [25, 26]. Patients with diffuse disease have higher rate of left internal mammary artery graft failure and poor prognosis in those undergoing CABG [27]. Similalrly, performing PCI produced less clinical benefits in patients with diffuse compared to focal coronary stenoses [26]. Therefore, patients with diffuse CAD are increasingly managed conservatively [21]. Notably, there is no angiographic consensus on the definition of CAD patterns, challenging any attempts to standardize clinical management in these patients.

Previous studies have highlighted the change in distal coronary pressure during pullback manoeuvre of the sensor mounted on the guidewire [24, 28]. Pressure loses along the coronary artery are related to numerous variables, including the presence of epicardial stenosis, length of the lesion, plaque features and their impact on laminar flow and viscous friction [24, 25, 26, 28, 29, 30]. Collectively, these factors would influence coronary pressure and produce a map of their integrated effects on pressure loses as well as epicardial resistance. Interestingly, mild epicardial coronary resistance was present in early stages of coronary atherosclerosis and was translated into myocardial ischaemia on positron emission tomography (PET) prior to segmental coronary stenosis on coronary angiography [24, 31]. The ability to standardise the relationship between changes in pressure over the length of the coronary artery would provide a functional assessment of the distribution of CAD. It may also overcome the current limitations of using coronary angiography for anatomical assessment of CAD patterns.

## 3. The Concept of PPG

**Fig. 1.**
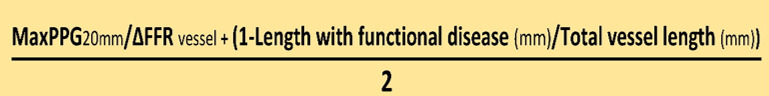
**Pullback pressure gradient calculation**. MaxPPG, maximum pullback pressure gradient; ΔFFR, The decrease in fractional flow reserve (FFR) after adenosine administration from baseline FFR value.

The integrated effects of plaque characteristics and flow patterns are not static features and change over the length of the coronary artery. Accurate evaluation of the correspondent changes in pressure loses in relation to the distribution of atherosclerosis requires steady pullback of the guidewire which was accomplished using motorized FFR pullback [21, 32]. This technique provided a new term of FFR drop, which allowed better understanding of the contribution of diffuse and/or focal CAD during pullback manoeuvre. By combining the magnitude of pressure drop alongside the extent of CAD, defined as the functional decrease in pressure over the entire length of the coronary artery, a new metric was devised to enable precise assessment of the spatial distribution of CAD [21]. PPG index is derived from FFR pullback using the above two factors of maximal pressure drop over 20 mm of the length of the coronary artery (functional magnitude of the disease) and the entire pressure drop of the full length of the coronary artery (functional extent of the disease) (Fig. 1). It provides a fully quantitative and more reproducible measure of the patterns of CAD compared to coronary angiography. PPG index can be established using manual pullback with excellent intra- and interobserver reproducibility [33].

In a proof-of-concept study PPG index reclassified more than one third of CAD patterns compared to coronary angiography [17, 21]. This reclassification was predominately from anatomical focal disease to combined or diffuse CAD. PPG index could be considered as a spectrum, that ranges from zero to one, to assess the focality or diffuseness of CAD, whereby the higher the index, the more focal the stenosis. Furthermore, PPG index allowed better characterization of patients with serial coronary lesions. These patients exhibited three distinct functional patterns related to the number of pressure drop during pullback manoeuvre [32]. PPG index not only was the highest among those patients with two focal pressure drops, but was able to independently predict the presence of this functional pattern among patients with diffuse CAD [32]. Such feature may assist with the suitability of coronary revascularization in patients with serial coronary lesions and help guiding coronary revascularization strategies in patients with various CAD patterns.

## 4. PPG Index and Ischemia

Several studies assessed the relationship between PPG index and coronary ischemia, measured using FFR [21, 34, 35]. Prior to PCI, there was an apparent disconnect between baseline FFR and PPG index [21, 34, 35]. In the validation study by Collet *et al*. [21] patients with the lowest tertile of PPG index also had the lowest FFR, despite having the most severe diameter stenosis [17]. A subsequent multicentre study highlighted that patients with focal lesions had lower FFR value which corresponded to a larger degree of diameter stenosis. The cohort of patients in the latter study were divided into two groups using a cut-off of >0.73 to define focal CAD [31, 35]. Using the same cut-off Ohashi *et al*. [34] reinforced this inconsistency between baseline FFR and PPG index [30]. In this randomised trial, patients with diffuse CAD who were assigned to FFR-guided PCI optimization strategy had lower pre-PCI FFR value compared to those with focal disease. In contrast, those with diffuse CAD who were assigned to standard practice had higher pre-PCI FFR compared to focal CAD. Of interest, across the three studies, the left anterior descending artery (LAD) was consistently less likely to demonstrate focal CAD pattern. This is unlikely to be related to an inherent pathophysiological process but directly linked to pressure loss phenomenon over the length of the LAD and its subsequent impact on measuring PPG index [25, 29].

Intuitively, the higher the PPG index, the more likely the potential gain in correcting epicardial resistance and achieving higher FFR post PCI. Recent studies have confirmed that patients with focal disease, defined as PPG index >0.73, achieved higher post PCI FFR compared to those with diffuse disease (0.91 ± 0.07 versus 0.86 ± 0.05, *p* < 0.001) [34, 35]. There was a relatively modest correlation between PPG index and post PCI FFR, highlighting CAD pattern as an additional mechanism of sub-optimal FFR results post PCI [30, 31, 32, 34, 35, 36]. Other mechanisms include sub-optimal stent results (under-expansion, edge dissection) and significant residual plaque burden outside the stented segment causing residual pressure gradient [34, 37]. Unsurprisingly, stent optimization had little value in relation to post PCI FFR in patients with diffuse disease [30, 34].

The change or delta FFR in response to PCI was also related to PPG index. Patients with focal disease had higher delta FFR compared to those with diffuse disease (0.33 ± 0.14 versus 0.17 ± 0.12, *p* < 0.001) [34, 35]. Importantly, the change in FFR was largely determined by PPG index at baseline ($R^{2}$ = 0.51, *p* < 0.001). This provides important clinical information that may guide clinical management, including insights into symptomatic relief and clinical outcomes post PCI. A combined data from Fractional Flow Reserve vs Angiography for Multivessel Evaluation (FAME) and FAME 2 with available pre- and post PCI FFR highlighted a significant association between symptomatic relief and delta FFR [34, 38]. Moreover, patients with delta FFR in the lowest tertile had twice the events rate of vessel oriented clinical outcomes compared to those with highest tertile [hazard ratio, 2.01] [95% confidence interval (1.03–3.92); *p* = 0.04] [34, 38].

The change in coronary flow reserve (CFR) post PCI followed the same patterns as the change in FFR in relation to PPG index [30, 34]. The delta CFR in patients with diffuse CAD was of smaller magnitude compared to patients with focal CAD following PCI [30, 34]. Additionally, the reduction in index microcirculatory resistance (IMR) following PCI was also of smaller magnitude in patients with diffuse compared to focal disease and final IMR was numerically, but not statistically, higher in patients with diffuse disease [30, 34]. Whether these changes at the level of microcirculation are related to plaque characteristics, beyond the role of residual disease in patients with diffuse CAD, warrant further studies.

## 5. PPG Index and Atherosclerotic Plaque Characteristics 

The interplay between CAD patterns, vessel hemodynamic and their impacts on plaque features were early recognized [35, 36, 37, 39, 40, 41]. The variations in local pressure gradient and its effect on laminar flow and wall shear stress is a recognised factor in the development and progression of coronary atherosclerotic plaque [37, 41]. Previous studies have shown that FFR was associated with vulnerable plaque and patients with lower FFR had more prevalent silent ruptured plaques [42, 43]. Nonetheless, direct evaluation of the impact of pressure loses on plaque characteristics was limited given the lack of longitudinal hemodynamic assessment on changes in plaque morphology.

The advent of PPG index allowed direct assessment of the regional relationship between plaque morphology and CAD patterns [40, 44]. Plaque burden, quantified across the entire coronary artery, was significantly higher in patients with diffuse disease compared to those with focal pattern (defined according to PPG index) [40, 44]. However, at the level of the minimal lumen area, this relationship was reversed and patients with diffuse CAD pattern had smaller plaque burden compared to individuals with focal disease [40, 44]. Likewise, there was a variation in plaque composition according to CAD patterns, as well as plaque volume. Using CT to characterise atherosclerotic plaque, patients with diffuse disease had more prevalent calcified plaques, whilst patients with focal CAD pattern had lower attenuation and non-calcified plaques [40, 44]. Invasive imaging modalities with optical coherence tomography (OCT) also revealed differential plaque characteristics in those with focal versus diffuse disease [44, 45]. Two studies revealed an association between high-risk plaque features and CAD patterns according to PPG index. The extent of lipid circumferential distribution was higher in patients with focal disease [40, 41]. The prevalence of thin-cap fibro-atheroma was also higher in patients with focal compared to diffuse disease [40, 41]. Finally, there was an inverse relationship between the thickness of fibrous cap and PPG index which translated into higher proportion of plaque ruptures in those with focal compared with diffuse disease [40, 41]. Such a differential relationship between CAD patterns and plaque morphology is also evident when assessing coronary hemodynamic. Metrics of shear wall stress were significantly higher in patients with focal compared to diffuse disease [41, 45]. Moreover, the combination of focal disease with high focal pressure gradient and high shear wall stress may synergistically contribute to destabilising plaque leading to acute plaque rupture and subsequent clinical events [41, 45].

**Table 1.** **Features of coronary artery disease according to the pullback pressure gradient**.

	Low PPG index	High PPG index
Invasive coronary angiography	Diffuse disease	Focal disease
	More likely with LAD	Less likely with LAD
Ischemia using FFR	Various at baseline	Various at baseline
	Low FFR post revascularization	High FFR post revascularization
	Delta FFR is of smaller magnitude	Delta FFR is of larger magnitude
Plaque burden	Large across the entire vessel	Small across the entire vessel
	Small at MLA level	Large at MLA level
Plaque characteristics	Calcified	Low-attenuation and non-calcified
	Low circumferential lipid distribution	High circumferential lipid distribution
Plaque stability	TCFA and sites of ruptured plaques less prevalent	TCFA and site of ruptured plaques more prevalent
Symptoms	Less benefits following revascularization	More benefits following revascularization

FFR, fractional flow reserve; LAD, left anterior descending artery; MLA, minimal lumen area; PPG, pullback pressure gradient; TCFA, thin cap fibroatheroma.

## 6. Clinical Outcomes according to PPG Index

Early studies highlighted the differential symptomatic response to PCI according to CAD patterns [42, 46]. Importantly, almost 1 in 4 patients remained symptomatic after PCI and this residual angina burden has been linked to worse prognosis, including cardiovascular mortality [43, 44, 45, 47, 48, 49]. These phenomenon were borne out using PPG index. A sub-analysis from the Trial of Angiography vs. pressure-Ratio-Guided Enhancement Techniques–Fractional Flow Reserve (TARGET-FFR) randomised clinical study included 107 patients who underwent baseline and follow up Seattle Angina Questionnaire (SAQ) to assess any difference in patient’s symptomatic status post PCI according to their CAD patterns [46, 50]. At baseline, the mean 7 items SAQ was comparable with no difference in the summary score among patients with focal versus diffuse CAD. Following PCI, patients with focal disease had significantly higher SAQ-7 summary score compared to patients with diffuse disease, with mean difference of 11.5 points [95% confidence interval (2.8 to 20.3), *p* = 0.01] [46, 50]. Such difference is of a very large magnitude when compared to the difference of merely 2.9 points in response to invasive revascularization on top of medical therapy in the International Study of Comparative Health Effectiveness with Medical and Invasive Approaches (ISCHEMIA) trial [47, 51]. Furthermore, freedom from angina was more frequent in patients with focal compared to diffuse disease (27.5% vs 51.9%; *p* = 0.020) [46, 50]. Remarkably, more than half of the patients with diffuse disease remained symptomatic despite receiving longer and more stents [46, 50]. Overall, PPG index may be used to guide clinicians as well as patients with regards to symptomatic benefits prior to performing PCI.

Data from Mizukami *et al*. [35] highlighted the difference in procedural outcomes according to PPG-defined CAD patterns [31]. Using OCT, the authors reported that patients with diffuse disease had significantly smaller stent area as well as smaller distal reference lumen area [31, 35]. Notably, the proximal reference lumen area was comparable between patients with focal versus diffuse disease, highlighting the current challenges in percutaneously revascularizing the latter group [31, 35]. Like the data from Collet *et al*. [50] the number and length of stents were larger in patients with diffuse disease which translated into higher post procedural cardiac biomarkers compared to patients with focal disease [35]. Moreover, the number of stent edge dissection was numerically more frequent in patients with diffuse compared to focal disease [10 (15.4%) vs. 2 (5.4%), *p* = 0.24] [31, 35].

Beyond procedural outcomes, PPG index was also shown to predict target vessel failure. At 2 years, the incidence of target vessel failure was more frequent in patients with diffuse disease compared to focal disease [48, 52]. It is important to highlight that the study by Shin *et al*. [52] calculated PPG index using quantitative flow ratio (QFR) [48]. QFR-derived PPG index is a well validated metric and has been highlighted as a useful adjunctive tool in performing coronary revascularization [53, 54]. Clinical outcome data using FFR-derived PPG index are currently studied in the PPG global registry and its results are eagerly awaited [51].

There are some limitations associated with the use of PPG and invasive ischemia assessment. Pressure derived FFR simulates relative coronary flow reserve but is not a direct measure or simulation of absolute coronary flow. FFR does not reflect or determine the size of left ventricle mass at risk, which is a major determinant of mortality risk and the likelihood of benefit from revascularization. While FFR is a major step toward physiologic severity beyond angiogram anatomy, it does not predict physiologic severity for patient selection for revascularization that improves survival probability. Whilst, PPG is considered the next substantial step in characterising CAD (focal versus diffuse), PPG remains limited as a pressure-based simulation of relative flow reserve and is subjected to the same limitations to FFR. Moreover, it might not be able to differentiate hyperemic pressure fall due to high coronary blood flow through mild diffuse CAD from flow limiting diffuse CAD. Finally, no trial of FFR, PPG or their anatomic simulations identifies patients for whom revascularization improves survival probability. In contrast, absolute rest and stress myocardial perfusion per pixel in mL/min/g, coronary flow reserve as their ratio and coronary flow capacity combing stress perfusion and CFR quantify artery specific size-severity of abnormalities that do predict sufficiently high mortality risk that is reduced by 54% to 43% after revascularization in large clinical cohorts followed over 10 years and in a randomized trial respectively [55, 56, 57, 58].

## 7. Conclusions

PPG index is a novel metric and an emerging tool that provide quantitative measure on the focality or diffuseness of CAD. It provides a precise description of the longitudinal distribution of atherosclerotic plaque and enables clinicians to predict symptomatic response, residual ischemia, and potentially target vessel failure in patients undergoing percutaneous revascularization.
